# Immunological Drug–Drug Interactions in Immune Checkpoint Inhibitor Therapy: Mechanisms, Clinical Evidence, and Artificial Intelligence

**DOI:** 10.1007/s11912-026-01789-0

**Published:** 2026-05-14

**Authors:** Chin Hang Yiu, Kevin Winardi, Christine Y. Lu

**Affiliations:** 1 The University of Sydney School of Pharmacy, Camperdown, Sydney, NSW Australia; 2 Kolling Institute, Faculty of Medicine and Health, The University of Sydney and the Northern Sydney Local Health District, St Leonards, Sydney, NSW Australia; 3 Laboratory of Ageing and Pharmacology, Kolling Institute, Faculty of Medicine and Health, The University of Sydney and the Northern Sydney Local Health District, St Leonards, Sydney, NSW Australia; 4 Department of Pharmacy, Royal North Shore Hospital, St Leonards, Sydney, NSW Australia

**Keywords:** Immune checkpoint inhibitors, Drug interactions, Artificial intelligence, Machine learning, Natural language processing

## Abstract

**Purpose of Review:**

Immune checkpoint inhibitors (ICIs) have transformed cancer therapy, producing durable responses across multiple malignancies. However, treatment outcomes may be influenced by immunological drug–drug interactions (DDIs) arising from commonly prescribed concomitant medications. Unlike classical pharmacokinetic or pharmacodynamic DDIs, these interactions operate through systemic mechanisms that modulate anti-tumour immunity, including alterations to the gut microbiome, immune signalling pathways, and the tumour microenvironment. This review proposes a conceptual framework for "immunological DDIs" (iDDIs), extending beyond metabolic interactions toward a system-level understanding of immune regulation.

**Recent Findings:**

We synthesise current evidence on commonly used medication classes—organised by their primary immunological mechanisms: (1) gut microbiome-mediated effects, (2) systemic immunosuppression, and (3) tumour microenvironment modulation—and their impact on ICI efficacy and safety. Meta-analyses suggest that certain medications, particularly antibiotics and proton pump inhibitors, are associated with poorer clinical outcomes, although confounding by indication and disease severity remain important limitations. Artificial intelligence (AI) is an emerging approach to detect and characterise complex DDIs using large-scale clinical and real-world data. Natural language processing, machine learning models, and large language models show potential for extracting medication exposure, predicting adverse events, and supporting clinical decision-making.

**Summary:**

Most AI applications remain at an early stage, with limited external validation and uncertain clinical utility. Future research should integrate mechanistic biology, prospective clinical studies, and explainable AI approaches to improve identification of iDDIs and optimise the safe and effective use of ICIs in oncology.

## Introduction

Cancer is the primary cause of death and premature mortality worldwide [1, 2]. Immune checkpoint inhibitors (ICIs) have transformed cancer therapy, producing durable responses across a range of malignancies [3]. These agents function by modulating the host immune system through inhibition of immune checkpoint pathways such as cytotoxic T-lymphocyte–associated protein-4 (CTLA-4) and the programmed cell death-1 (PD-1)/programmed cell death-ligand 1 (PD-L1) axis [4]. Currently approved ICIs include anti-PD-1 antibodies (e.g., pembrolizumab, nivolumab, cemiplimab, dostarlimab), anti-PD-L1 antibodies (e.g., atezolizumab, durvalumab, avelumab), and the anti-CTLA-4 antibody (ipilimumab). In 2022, the approval of relatlimab has expanded this therapeutic class to include lymphocyte-activation gene 3 (LAG-3) inhibition, further advancing the immunotherapy landscape [5]. Despite these advances, real-world effectiveness remains variable and may be influenced by polypharmacy and the complexity of drug–drug interactions (DDIs).

Traditionally, DDIs have been conceptualised within pharmacokinetic (PK) and pharmacodynamic (PD) frameworks, with a focus on metabolic interactions mediated by drug-metabolizing enzymes and transmembrane transporters, such as cytochrome P450 (CYP450) systems [6]. However, ICIs differ fundamentally from conventional therapies. As monoclonal antibodies, they are primarily cleared via proteolytic degradation and exhibit prolonged half-lives, rendering classical PK-based interactions less relevant [7]. Instead, ICIs give rise to a distinct class of interactions, termed immunological DDIs (iDDIs) that operate through host-mediated mechanisms affecting anti-tumour immunity [8]. These include modulation of immune recognition, alterations in the gut microbiome, and changes within the tumour microenvironment (TME) [8, 9].

The clinical importance of iDDIs is increasingly recognised, as concomitant medications emerge as potentially modifiable determinants of ICI efficacy and safety [10]. However, their identification and interpretation in routine practice remain challenging. Relevant exposure data are often embedded within large-scale, unstructured clinical records, and the effects of concomitant medications are highly context-dependent, influenced by factors such as timing, indication, baseline inflammation, and microbial diversity [11]. Interpretation is further complicated by potential confounding; for example, medication use may reflect comorbidities and greater disease severity rather than directly mediating reduced treatment efficacy. This high-dimensional complexity limits the utility of traditional analytical approaches and necessitates the integration of artificial intelligence (AI)–driven frameworks capable of modelling non-linear, temporally dynamic interactions. AI methods enable the integration and analysis of large volumes of structured and unstructured data from electronic medical records, administrative claims databases, and other real-world data sources, providing new opportunities to detect complex patterns associated with treatment outcomes and adverse events [12, 13]. To address these challenges, this review integrates mechanistic and clinical perspectives on immunological DDIs while outlining the emerging role of AI in their identification and management. We begin by outlining the immunological basis of ICI therapy, before examining how concomitant medications may disrupt the immune equilibrium through three principal mechanisms: gut microbiome dysbiosis, systemic immunosuppression, and TME modulation.

## Immunological Basis of ICI Therapy and the Immune Balance

The efficacy and safety of ICIs are fundamentally rooted in the biology of adaptive immunity. T-lymphocytes, the primary effectors of ICI-mediated anti-tumour responses, undergo a carefully regulated developmental trajectory. Notably, thymic function itself is emerging as a clinically relevant and potentially modifiable determinant of ICI response and health longevity [14, 15]. Haematopoietic stem cells in the bone marrow give rise to T-cell progenitors, which migrate to the thymus for maturation and selection. Within the thymus, developing T cells undergo positive and negative selection to establish a repertoire capable of recognising foreign antigens while maintaining tolerance to self-antigens [16]. Following thymic education, mature naïve T cells enter the peripheral circulation, where they may be activated upon encounter with tumour-associated antigens presented by antigen-presenting cells in the context of appropriate co-stimulatory signals.

**Fig. 1:**
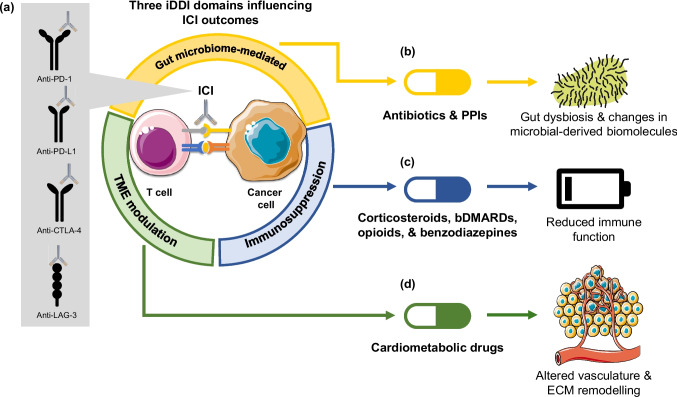
Conceptual framework of immunological drug–drug interactions (iDDIs) during immune checkpoint inhibitor (ICI) therapy. **a** ICIs — including anti-PD-1, anti-PD-L1, anti-CTLA-4, and anti-LAG-3 antibodies — restore anti-tumour T-cell activity by releasing immune checkpoint pathways at the interface between T cells and cancer cells. Concomitant medications may disrupt this immune equilibrium through three principal mechanistic domains. **b** Gut microbiome–mediated iDDIs: antibiotics and proton pump inhibitors (PPIs) induce microbial dysbiosis and alter microbial-derived biomolecules that support anti-tumour immune priming. **c** Systemic immunosuppression–mediated iDDIs: corticosteroids, biologic disease-modifying antirheumatic drugs (bDMARDs), opioids, and benzodiazepines suppress immune cell function, reducing the T-cell–mediated anti-tumour responses required for effective checkpoint blockade. **d** Tumour microenvironment (TME) modulators: cardiometabolic drugs (e.g., metformin, RAAS inhibitors, statins) may reshape the TME through altered vasculature and extracellular matrix (ECM) remodelling, indirectly influencing immune cell infiltration. The direction and clinical significance of each interaction are summarised in Table 1

Immune checkpoint pathways, including CTLA-4, PD-1/PD-L1, and LAG-3, serve as critical regulators of this activation process, functioning as “brakes” that prevent excessive immune activation and maintain immunological homeostasis (Fig. 1a) [4, 17]. As one of the hallmarks of cancer, tumours exploit these inhibitory pathways to evade immune surveillance, and ICIs function by releasing these brakes, thereby restoring anti-tumour T-cell activity [18, 19]. However, this therapeutic mechanism creates an inherent tension—a narrow immunological window within which clinical benefit must be balanced against the risk of immune-related toxicity. Excessive immune activation, driven by unrestrained T-cell responses in the absence of checkpoint regulation, manifests as immune-related adverse events (irAEs) affecting virtually any organ system [20]. Conversely, insufficient restoration of immune activity—whether due to tumour-intrinsic resistance mechanisms or host-extrinsic factors such as concomitant medications—results in reduced therapeutic efficacy.

This conceptual framework—balancing immune activation against immune tolerance—provides the foundation for understanding how concomitant medications may act as iDDIs. Medications that suppress immune function which may accelerate thymic involution (e.g., corticosteroids, opioids) risk tipping the balance toward reduced efficacy, while those that alter the immune system indirectly (e.g., antibiotics disrupting the gut microbiome) may compromise the immune priming required for optimal checkpoint blockade (Fig. 1). The following sections examine how specific concomitant medication classes may disrupt this immunological equilibrium, organised by their primary mechanism of interaction.

## Immunological Drug–Drug Interactions with Immune Checkpoint Inhibitors

### Gut Microbiome–Mediated iDDIs: Antibiotics and Proton Pump Inhibitors

The gut microbiome represents the most extensively characterised pathway through which concomitant medications may indirectly influence ICI efficacy (Fig. 1b). A diverse and intact gut microbial ecosystem supports anti-tumour immunity through multiple mechanisms, including dendritic cell activation, CD8 + T-cell priming, and modulation of systemic inflammatory tone [21]. Two of the most prescribed medication classes—antibiotics and proton pump inhibitors (PPIs)—converge on this pathway through distinct but complementary mechanisms of microbiome disruption.

#### Antibiotics

Antibiotics represent the most extensively studied example of iDDIs during ICI therapy. Preclinical studies have demonstrated that specific commensal bacterial species—including *Bifidobacterium*, *Akkermansia muciniphila*, and members of the Ruminococcaceae family—are enriched in ICI responders and are associated with enhanced CD8 + and CD4 + T-cell effector function and dendritic cell activation [22–24]. Antibiotic exposure can induce gut microbial dysbiosis, reducing microbial diversity and depleting bacterial taxa that support anti-tumour immunity, thereby impairing immune priming and attenuating the efficacy of immune checkpoint blockade.

Multiple meta-analyses consistently demonstrate an association between peri-ICI antibiotic exposure and poorer clinical outcomes. Huang et al. reported, in a pooled analysis of 2,740 patients across 19 studies, that antibiotic use was associated with reduced overall survival (OS) across multiple malignancies, including non-small cell lung cancer (NSCLC), renal cell carcinoma (RCC), and urothelial carcinoma [25]. The effect was particularly significant in NSCLC, where antibiotic exposure was associated with markedly worse survival (hazard ratio [HR] = 2.68, 95% confidence interval [CI] 2.19–3.28). These findings are supported by a larger meta-analysis by Crespin et al. encompassing 107 studies, which demonstrated a pooled HR of 1.61 (95% CI 1.48–1.76) for OS among patients exposed to antibiotics around ICI initiation [26]. A critical determinant of this interaction is the timing of exposure, with the strongest negative effects observed when antibiotics are administered within 30-90 days prior to ICI initiation [26, 27]. The spectrum of antibiotic exposure may also be relevant, as broad-spectrum antibiotics have been associated with significantly lower response rates and shorter PFS compared with narrow-spectrum agents, consistent with a greater degree of microbiome disruption [28]. Although similar associations have been reported in patients receiving chemoimmunotherapy, the magnitude of effect appears attenuated, potentially reflecting partial compensation by the cytotoxic component [29, 30]. Beyond efficacy, antibiotic exposure has also been linked to altered risk of irAEs, including higher rates of pneumonitis and thyroid irAEs among patients receiving anti-PD-1/PD-L1 therapy [31].

Despite the consistency of these findings, causal inference remains limited by the observational nature of the available evidence. Retrospective studies are particularly susceptible to confounding by indication [32, 33], as patients receiving antibiotics often have underlying infections that independently predict poorer clinical outcomes. This introduces a ‘sick-user’ bias, whereby antibiotic exposure may serve as a surrogate marker of disease severity or systemic inflammation rather than a direct mediator of reduced ICI efficacy. Given the ethical infeasibility of randomized controlled trials (RCTs) in which antibiotics are deliberately administered, well-designed prospective observational studies are required to better account for infection severity, antibiotic class, and treatment context.

#### Proton Pump Inhibitors

Similar to antibiotics, PPIs may modulate ICI outcomes through microbiome-mediated mechanisms. PPIs are among the most commonly prescribed medications worldwide, with nearly one in four patients with cancer receiving concomitant PPI therapy [34, 35]. By increasing gastric pH, PPIs can alter the composition and diversity of the gut microbiota, including shifts in bacterial taxa that may influence anti-tumour immune responses and the efficacy of immune checkpoint blockade [36–38].

Meta-analytic evidence generally supports a negative association between PPI use and ICI outcomes [39–41]. A large meta-analysis of 41 studies involving over 20,000 patients reported that PPI use was associated with worse OS (HR 1.37, 95% CI 1.23–1.52) and PFS (HR 1.28, 95% CI 1.15–1.42) [39]. These negative associations were observed across multiple tumour types including NSCLC and urothelial cancer and were most pronounced when PPIs were administered at baseline or within 60 days before ICI initiation. A more recent meta-analysis by Ciappina et al. confirmed these findings, documenting an 18% increased mortality risk with PPI exposure (HR 1.18, 95% CI 1.11–1.25) [41].

As with antibiotics, the observational nature of the current evidence limits causal inference [32, 33]. Residual confounding by underlying disease characteristics may contribute to the observed associations; for example, patients requiring PPIs may have comorbidities or disease features that independently predict poorer outcomes [42]. Well-designed prospective studies are therefore required to disentangle direct microbiome-mediated effects from confounding clinical factors and to clarify the true impact of PPI exposure on ICI efficacy. Collectively, these findings highlight the central role of the gut microbiome in mediating iDDIs and provide a conceptual foundation for understanding similar interactions across other commonly prescribed medications.

### Systemic Immunosuppression–Mediated iDDIs: Corticosteroids, Immunomodulators, Opioids, and Benzodiazepines

A second category of iDDIs involves medications that may attenuate systemic immune function, potentially compromising the T-cell–mediated anti-tumour responses that underpin ICI efficacy (Fig. 1c). Corticosteroids are established immunosuppressive agents with well-characterised effects on T-cell proliferation, cytokine production, and effector function. Opioids and benzodiazepines, while not classified as immunosuppressants in conventional pharmacology, are included in this section because their proposed iDDI mechanisms operate through modulation of immune cell function, rather than microbiome or TME modulation.

#### Corticosteroids

Corticosteroids are among the most frequently co-prescribed medications in immuno-oncology, used for symptom palliation, management of brain metastases, and treatment of irAEs [43]. Their broad immunosuppressive properties—including inhibition of T-cell proliferation, suppression of effector cytokine production, and promotion of regulatory T-cell activity—raise mechanistic concerns regarding attenuation of ICI efficacy [44].

Clinical evidence linking corticosteroid use to ICI outcomes is complex and appears to depend largely on the indication for steroid use. Arbour et al. and Ricciuti et al. were among the first to report that baseline corticosteroid use at doses of ≥ 10 mg prednisone equivalent per day was associated with inferior PFS and OS in NSCLC patients receiving ICIs [45, 46]. This illustrates a key concept we term ‘indication-inversion,’ whereby the apparent effect of a drug on ICI outcome is driven predominantly by the clinical context in which it is prescribed rather than its direct immunological action. Specifically, Ricciuti et al. demonstrated that the negative prognostic effect was primarily confined to patients receiving corticosteroids for palliative indications (e.g., brain metastases, dyspnoea), suggesting that the underlying disease burden, rather than immunosuppression per se accounted for poorer outcomes [46]. Subsequent systematic reviews and meta-analyses have confirmed this pattern, indicating that adverse survival outcomes are primarily associated with corticosteroid use for supportive or palliative care, whereas corticosteroids administered for irAE management do not appear to compromise survival [47, 48].

Emerging evidence further suggests that this relationship is nuanced. A post hoc analysis of six clinical trials involving 834 patients treated with combined anti–PD-1 and anti–CTLA-4 therapy found that higher peak corticosteroid doses for irAE management were independently associated with worse PFS (HR 1.43 for 2 vs. 0.5 mg/kg) and OS (HR 1.66 for 2 vs. 0.5 mg/kg), whereas cumulative corticosteroid exposure was not significantly associated with survival [49]. These findings suggest that peak corticosteroid dosing intensity, rather than total cumulative exposure, may influence ICI outcomes, highlighting the potential benefit of initiating irAE therapy at lower corticosteroid doses when clinically feasible. Furthermore, recent evidence suggests that inhaled corticosteroids for respiratory comorbidities such as chronic obstructive pulmonary disease do not appear to compromise ICI efficacy [50], suggesting that the importance of both route and systemic exposure in modulating immune interactions. This bimodal, context-dependent effect, where the clinical indication can invert a drug’s apparent prognostic significance, poses a significant challenge for traditional rule-based interaction screening. It further highlights the need for context-aware analytical frameworks, including AI-driven approaches, to accurately capture and predict the impact of corticosteroid exposure on ICI outcomes (Section "Artificial Intelligence Approaches for Identifying and Managing Immunological DDIs").

#### Immunomodulators in the context of pre-existing autoimmune disease

Cytokines such as tumour necrosis factor (TNF) and interleukin (IL) have been implicated in both anti-tumour immunity and immune-related toxicity during ICI therapy, raising the question of whether targeted cytokine blockade could modulate ICI outcomes [51, 52]. This is particularly relevant for patients with pre-existing autoimmune diseases who require biologic disease-modifying antirheumatic drugs (bDMARDs) or other immunomodulatory agents. Although clinical trials have historically excluded patients with active autoimmune conditions, an emerging paradigm proposes that targeted cytokine inhibition—particularly TNF or IL-6 blockade— may confer synergistic anti-tumour efficacy while mitigating irAE toxicity [53]. Consistent with this hypothesis, infliximab administered for the management of irAEs does not appear to compromise ICI efficacy [54, 55]. Nonetheless, the current evidence base remains limited to retrospective analyses and case reports, and prospective studies are needed to clarify the impact of specific immunomodulatory regimens on ICI outcomes in this patient population.

#### Opioids and benzodiazepines

Opioids are widely used for cancer-related pain management. Emerging evidence suggests their use may be associated with poorer outcomes in patients receiving ICI therapy, including reduced OS and PFS [56]. Although mechanisms remain incompletely understood, opioids are thought to exert immunosuppressive effects that could interfere with antitumour immune responses [57]. These effects may include suppression of natural killer cell cytotoxicity, impaired T-cell proliferation, and altered cytokine signalling, all of which could attenuate the immune activation required for effective checkpoint blockade. As with corticosteroids, confounding by indication is a key limitation, since opioid use is closely associated with advanced disease, pain burden, and overall functional decline.

Benzodiazepines are frequently prescribed in oncology for anxiety, insomnia, and procedural sedation [58]. Emerging preclinical evidence suggests that benzodiazepines may exert immunomodulatory effects through gamma-aminobutyric acid (GABA) receptor signalling on immune cells, including reduced T-cell proliferation, decreased pro-inflammatory cytokine production, and altered macrophage function [58]. While clinical data on the impact of benzodiazepine use during ICI therapy remain limited, their widespread use in cancer populations and biologically plausible immunosuppressive mechanisms warrant further investigation as potential contributors to systemic immunosuppression-mediated iDDIs [59–61]. Prospective studies are needed to determine whether benzodiazepines should be considered alongside corticosteroids and opioids in the assessment of immune-modulatory polypharmacy.

### Indirect Tumour Microenvironment Modulators

A third category of iDDIs comprises medications that may modulate anti-tumour immunity indirectly through effects on the tumour microenvironment (TME) (Fig. 1d). Unlike the direct immunosuppressive agents discussed above, these drugs—including metformin, renin–angiotensin–aldosterone system (RAAS) inhibitors, statins, and other cardiovascular agents—act through metabolic, vascular, or inflammatory pathways that shape the immune landscape within and around the tumour.

#### Metformin

Metformin has attracted considerable interest due to preclinical data indicating potential enhancement of PD-1 blockade through reduced tumour hypoxia and activation of the AMPK pathway [62]. However, a meta-analysis of 22 retrospective studies found that concomitant metformin use did not improve clinical outcomes in ICI-treated patients and may even correlate with inferior prognoses in certain populations (e.g., European population) [63]. These findings suggest that the immunomodulatory effects observed in preclinical models may not consistently translate into clinical benefit, possibly due to confounding by diabetes status or other comorbidities.

#### Angiotensin-converting Enzyme Inhibitors and Angiotensin Receptor Blockers

RAAS inhibitors, including angiotensin-converting enzyme (ACE) inhibitors and angiotensin receptor blockers (ARBs), have been investigated for their indirect effects on the TME [64]. Angiotensin II promotes immunosuppressive pathways, whereas RAAS inhibition may facilitate T-cell infiltration and reduce myeloid-derived suppressor cell activity [65]. A meta-analysis of 12 retrospective studies reported a potential survival benefit with concomitant RAAS inhibitor use during ICI therapy, with pooled HR of 0.85 (95% CI 0.75–0.96) for OS and 0.91 (95% CI 0.76–1.09) for PFS [66]. The effect appeared particularly significant in patients with urothelial carcinoma (HR 0.53, 95% CI 0.31–0.89) and RCC (HR 0.56, 95% CI 0.37–0.84) with respect to OS. However, these findings are derived solely from retrospective analyses and remain susceptible to residual confounding.

#### Other Cardiovascular and Metabolic Agents

Other cardiovascular medications—including statins, aspirin, and beta-blockers—have also been proposed as potential modulators of antitumour immunity, but the evidence remains limited and largely exploratory [67, 68]. For instance, statins may influence the TME through modulation of cholesterol metabolism in T cells and downregulation of PD-L1 expression [69]. NSAIDs, through COX-2 inhibition, may reduce immunosuppressive prostaglandin E₂ (PGE₂) signalling within the TME [70]. Beta-blockers may attenuate catecholamine-driven immunosuppression, which has been implicated in tumour immune evasion [71]. The sheer number of potential interactions across these drug classes generates substantial "clinical noise", highlighting the need for computational and AI-based approaches capable of integrating non-linear, high-dimensional data. Prospective clinical trials are necessary to determine whether these medications exert synergistic effects when combined with ICIs before they can be recommended as adjunctive therapies in routine practice. The key drug classes, proposed mechanisms, and clinical evidence across all three domains are summarised in Table 1.

**Table 1:** Concomitant medications associated with immunological drug–drug interactions (iDDIs) during immune checkpoint inhibitor therapy

Drug class	Proposed mechanism	Direction of association & key modifiers based on clinical evidence
Domain 1: Gut Microbiome–Mediated iDDIs (Section "Gut Microbiome–Mediated iDDIs: Antibiotics and Proton Pump Inhibitors")		
Antibiotics	Gut dysbiosis; reduced microbial diversity; depletion of immunostimulatory taxa; impaired DC activation and T-cell priming [21]	**Negative** (↓ OS, PFS, ORR) [25] [26, 27] **Timing:** 30–90 days pre-ICI = strongest association [26, 27] **Spectrum:** broad-spectrum antibiotics were associated with worse ICI outcomes [28] **Confounding:** “sick-user” bias [32, 33]
PPIs	Gastric pH elevation → altered gut microbiota composition and ↓ diversity; possible direct immunomodulatory effects [36–38]	**Negative** (↓ OS, PFS) [39–41] **Timing:** strong association if administered at baseline or within 60 days pre-ICI initiation [39] **Confounding:** comorbidity burden [42]
Domain 2: Systemic Immunosuppression–Mediated iDDIs (Section "Systemic Immunosuppression–Mediated iDDIs: Corticosteroids, Opioids, and Benzodiazepines")		
Corticosteroids	↓ T-cell proliferation, ↓ effector cytokines, ↑ Tregs; selective depletion of memory CD8 + T cells [44]	**Context-dependent (**↓ OS/PFS in palliative indications; no effect in irAE indications) [47, 48] **Dose:** higher peak dose might be associated with ↓ OS/PFS, while cumulative corticosteroid exposure has no association [49] **Route:** inhaled steroids do not affect survival [50]
Biologics/bDMARDs (TNF inhibitors, IL antagonists)	TNF/IL-6 inhibition [51]; broad immunosuppression but emerging evidence of potential decoupling of efficacy from irAE toxicity	**Potentially beneficial** Targeted biologics (e.g., infliximab, tocilizumab) may not compromise efficacy; emerging paradigm of synergistic effects with ICIs while mitigating immune-related toxicity [53]
Opioids	↓ NK cell cytotoxicity; impaired T-cell proliferation; modulation of cytokine signalling [57]	**Negative** (↓ OS, PFS) [56] **Confounding:** proxy for pain burden and disease severity
Benzodiazepines	GABA receptor signalling on immune cells; possible ↓ T-cell proliferation, ↓ pro-inflammatory cytokines [58]	**Potentially negative** (↓ OS); not fully investigated with limited clinical data [59–61]
Domain 3: Indirect Tumour Microenvironment Modulators (Section "Indirect Tumour Microenvironment Modulators")		
Metformin	AMPK activation; ↓ tumour hypoxia; enhanced PD-1 blockade in preclinical models [62]	**No clinical benefit** [63] **Confounding:** diabetes severity and comorbidities
ACEi/ARBs	RAAS inhibition within TME; ↓ angiotensin II signalling; ↑ T-cell infiltration; ↓ MDSC activity [65]	**Potentially beneficial** (↑ OS) [66] **Tumour type:** strongest association observed in UC and RCC [66]
Statins, NSAIDs, Beta-blockers	Statins: ↓ PD-L1 [69]; NSAIDs: COX-2 inhibition, ↓ PGE₂ [70]; Beta-blockers: ↓ catecholamine immunosuppression [71]	**Potentially beneficial with statins** (↑ OS, PFS); **No clinical benefit with beta-blockers/NSAIDs (except low-dose aspirin)** [67, 68]

Abbreviations: *ACEi* angiotensin-converting enzyme inhibitor, *AMPK* AMP-activated protein kinase, *ARB* angiotensin receptor blocker, *bDMARDs* biologic disease-modifying antirheumatic drugs, *COX* cyclooxygenase, *DC* dendritic cell, *GABA* gamma-aminobutyric acid, *ICI* immune checkpoint inhibitor, *iDDI* immunological drug–drug interaction, *IL* interleukin, *irAE* immune-related adverse event, *MDSC* myeloid-derived suppressor cell, *NK* natural killer, *NSAID* non-steroidal anti-inflammatory drug, *ORR* objective response rate, *OS* overall survival, *PFS* progression-free survival, *PGE*₂ prostaglandin E₂, *PPI* proton pump inhibitor, *RAAS* renin–angiotensin–aldosterone system, *RCC* renal cell carcinoma, *TME* tumour microenvironment, *TNF* tumour necrosis factor, *Treg* regulatory T cell, *UC* urothelial carcinoma. Reference numbers correspond to the main manuscript

## Artificial Intelligence Approaches for Identifying and Managing Immunological DDIs

The clinical evidence detailed in Section "Immunological Drug–Drug Interactions with Immune Checkpoint Inhibitors" highlights a fundamental challenge: iDDIs are not static or isolated events, but dynamic, multi-dimensional processes that depend on timing, indication, and patient-specific context. The temporal sensitivity of antibiotic exposure, the indication-dependent effects of corticosteroids, and the microbiome-mediated associations observed with PPIs illustrate the limitations of conventional analytical approaches and require methods capable of capturing longitudinal dynamics, contextual clinical reasoning, and high-dimensional biological interactions. These challenges motivate AI as a complementary framework for detecting, modelling, and managing iDDIs by integrating multi-modal clinical and biological data while accounting for temporal dynamics and confounding factors. Unlike classical drug–drug interactions, which are typically mediated by enzyme inhibition or induction and operate over short timeframes, iDDIs involve microbiome modulation, TME alterations, and broader immune system effects that may evolve over weeks to months. AI-based approaches enable integration of multi-modal data—including clinical records, medication exposure, and biological features—while accounting for temporal complexity and confounding factors such as the “sick-user” bias**.** However, AI applications for identifying and managing iDDIs remain at an early stage of development, with current methods primarily demonstrating exploratory or proof-of-concept utility rather than established clinical implementation.

### Natural Language Processing–Based Approaches for DDI Extraction from Clinical Text

A substantial portion of clinically relevant information about concomitant medication use is embedded in unstructured clinical narratives, including physician notes and discharge summaries [72]. This is particularly important for iDDIs, where critical windows of exposure—such as the 30-day pre-ICI period for antibiotics—are often documented in text rather than structured pharmacy codes. Natural language processing (NLP) enables extraction of clinically relevant information from unstructured text at scale. NLP approaches range from rule-based systems to transformer-based deep learning models. Models such as Bidirectional Encoder Representations from Transformers (BERT) and its biomedical variants (BioBERT, ClinicalBERT, PharmBERT) excel at relation extraction and named entity recognition, achieving F1-scores exceeding 83% on benchmark DDI datasets [73–75] [76]. Hybrid architectures combining BERT embeddings, bidirectional long short-term memory (BiLSTM) networks, and Graph Attention Networks (GAT) further enhance performance, achieving precision of 81.8% and F1-scores of 82.5% [77].

In oncology, NLP-based pipelines have successfully extracted irAEs and temporally linked concomitant medication exposures from clinical notes [78, 79]. This capability is particularly relevant for temporally sensitive iDDIs, such as antibiotic exposure preceding ICI initiation. Challenges remain, including the need for domain-specific annotation, limited availability of labeled datasets, variability across electronic health record (EHR) systems, and limited external validation. However, NLP primarily supports structured extraction of medication exposure and temporal relationships and does not provide higher-level clinical reasoning, motivating generative approaches.

### Large Language Models for DDI Summarization and Clinical Decision Support

Building on these extraction capabilities, large language models (LLMs) provide higher-level reasoning and synthesis, enabling integration of complex pharmacological and clinical information. LLMs have created new opportunities for drug interaction analysis in oncology [80]. LLMs, including GPT-4 and domain-adapted variants such as DrugGPT and MEREDITH, can synthesise complex evidence into clinically coherent summaries and support context-dependent clinical decision-making. LLMs, such as DrugGPT, leverage curated clinical knowledge bases (e.g., DrugBank and clinical guidelines) to provide evidence-based recommendations for drug interactions and adverse events, outperforming general-purpose LLMs on pharmacovigilance tasks [81]. Oncology-specific applications utilise retrieval-augmented generation (RAG) to support treatment decisions, achieving high concordance with expert oncologist recommendations [82]. LLMs are well suited to capturing context-dependent distinctions central to iDDIs, including differentiating corticosteroid use for palliative indications versus irAE management.

Despite these advances, significant limitations remain. LLMs are prone to hallucination—generating plausible but factually incorrect content—which is particularly problematic in clinical pharmacology [83]. Current applications are often deployed without training on real-world clinical data or rigorous validation of their clinical benefit [84]. While LLMs are effective for knowledge synthesis and decision support, quantitative prediction of patient outcomes requires structured modelling approaches, motivating the use of machine learning techniques.

### Machine Learning for Predictive Modelling of ICI Outcomes

Machine learning (ML) enables integration of concomitant medication profiles with clinical, laboratory, and genomic features to predict ICI efficacy and safety. Traditional ML algorithms—including random forests, gradient-boosted trees (e.g., XGBoost), and support vector machines—havey single data modality alone been applied to structured EHR and administrative data to model treatment response and adverse events [78, 85]. These approaches can simultaneously adjust for multiple baseline prognostic factors, helping address confounding by indication.

ML has been used to identify patients at high risk for ICI–related adverse events, such as adrenal insufficiency or acute kidney injury (AKI), based on concomitant medication exposure (e.g., antibiotics and PPIs) [86, 87]. This is particularly relevant for disentangling confounded associations observed with medications such as PPIs, where underlying patient characteristics may influence outcomes.

Deep learning approaches, including Recurrent Neural Networks (RNNs) and Long Short-Term Memory (LSTM) networks, are well-suited for modelling longitudinal medication exposure and temporal relationships with ICI outcomes [88]. Time-series modelling captures dynamic vulnerability windows and critical exposure periods during therapy [26, 27, 39]. The clinical deployment of these predictive models, however, depends on their interpretability and trustworthiness, necessitating explainable AI approaches.

### Clinical Decision Support Systems and Explainable AI

Rule-based DDI alert systems are already embedded within most electronic medical record platforms and are routinely used to flag classical PK and PD interactions at the point of prescribing [89]. However, these systems rely on predefined drug-pair rules and are not designed to capture the context-dependent, temporally dynamic nature of iDDIs [90]. No equivalent alerting framework currently exists for immunological interactions. The translation of ML predictions into clinical practice requires clinical decision support systems (CDSSs) that are transparent, interpretable, and trustworthy [91]. Explainable artificial intelligence (XAI) methods, including SHapley Additive exPlanations (SHAP) and Local Interpretable Model-agnostic Explanations (LIME), are widely used in biomedical AI for post hoc interpretability [92–95].

In immunotherapy, SHAP analysis has identified predictive features of ICI response, such as exhausted T-cell signatures, across diverse cancer types [96]. Integrating medication history into XAI frameworks enables patient-specific reasoning—for example, clarifying whether a reduced response probability is driven by corticosteroid dose intensity or the timing of prior antibiotic exposure. Counterfactual explanations, such as “What would the predicted outcome be if this patient had not received antibiotics?”, further enhance interpretability [97].

Regulatory frameworks for AI-based CDSSs are evolving [98]. Systems targeting iDDIs will likely require demonstration of clinical utility, robust external validation, and transparent documentation of limitations. The integration of XAI methods into these systems is critical for ensuring that predictions are based on biologically plausible mechanisms rather than spurious correlations.

### Emerging Technologies: Omics and Knowledge Graphs

Beyond the established AI methods discussed above, several emerging technologies hold particular promise for advancing the mechanistic understanding of iDDIs. Spatial and single-cell omics technologies are transforming our ability to characterise the tumour immune microenvironment [99]. These technologies enable mapping of immune cell states, spatial organisation of T-cell populations, and identification of immunosuppressive niches potentially influenced by concomitant medication exposure. Spatial profiling could reveal whether antibiotic-induced microbiome dysbiosis alters the spatial distribution of tumour-infiltrating lymphocytes or whether corticosteroid exposure selectively depletes specific T-cell subsets within the TME. Further, a new-generation spatial multi-omics approach, which jointly profiles transcriptomics, metabolomics, and drug level, reveals regions of drug resistance due to spatial constraints and how local drug exposure may induce metabolic rewiring in a preclinical medulloblastoma model treated with palbociclib [100]. Given the importance of microbiome in ICI response, metaproteomics which captures both microbial- and host-associated proteins provides a powerful avenue to interrogate drug-microbe interactions. Recently, a high-throughput metaproteomics platform profiling over 300 drugs demonstrated that specific drug classes can reshape microbiome functional dynamics, while also highlighting substantial heterogeneity in drug responses across individuals [101].

Knowledge graphs (KGs) represent another promising avenue for integrating heterogeneous data sources into unified representations of biological and clinical knowledge. By structuring relationships between drugs, molecular targets, immune pathways, microbial taxa, and clinical outcomes, KGs can support hypothesis generation and mechanistic reasoning about iDDIs that would be difficult to achieve through any single data modality alone [102–104]. When integrated with ML models, KGs enable graph-based reasoning that can identify previously unrecognised interaction patterns—for example, linking a concomitant medication to downstream immune pathway effects through intermediary molecular nodes. The combination of spatial and single-cell omics with knowledge graph-based reasoning and clinical real-world data represents a frontier for mechanistic pharmacovigilance, potentially enabling the integration of molecular-level evidence into AI-driven clinical decision-making frameworks for iDDI detection and management.

**Fig. 2:**
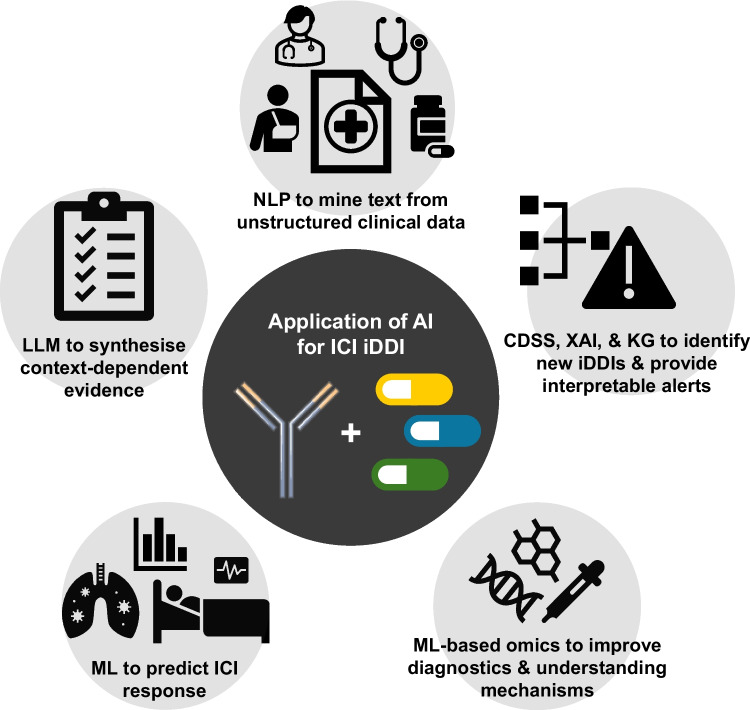
Artificial intelligence approaches for identifying and managing immunological drug–drug interactions (iDDIs) during immune checkpoint inhibitor (ICI) therapy. Five complementary AI domains address the central challenge of detecting and characterising iDDIs in patients receiving ICIs with concomitant medications. (1) Natural language processing (NLP) enables extraction of medication exposures and temporal relationships from unstructured clinical text (Section "Natural Language Processing–Based Approaches for DDI Extraction from Clinical Text"). (2) Large language models (LLMs) support context-dependent reasoning and evidence synthesis, such as distinguishing corticosteroid use for palliative indications versus immune-related adverse event management (Section "Large Language Models for DDI Summarization and Clinical Decision Support"). (3) Machine learning (ML) and deep learning approaches integrate clinical, medication, and biological features to predict ICI outcomes and adverse events (Section "Machine Learning for Predictive Modelling of ICI Outcomes"). (4) Clinical decision support systems (CDSSs), explainable AI (XAI), and knowledge graphs (KGs) enable interpretable, mechanism-informed alerts for clinical translation (Section "Clinical Decision Support Systems and Explainable AI"–"Emerging Technologies: Omics and Knowledge Graphs"). (5) ML-based omics technologies — including spatial transcriptomics, single-cell analysis, and metaproteomics — advance mechanistic understanding of drug–immune interactions within the tumour microenvironment (Section "Emerging Technologies: Omics and Knowledge Graphs")

Table 2 summarises the key distinctions between classical DDIs and iDDIs and outlines how each AI approach addresses specific challenges posed by immunological interactions. An overview of these five AI domains and their respective roles in iDDI detection and management is also presented in Fig. 2.

**Table 2:** Comparison of classical pharmacokinetic/pharmacodynamic drug–drug interactions (DDIs), immunological DDIs (iDDIs), and potential corresponding AI/ML approaches

Feature	Classical DDI (PK/PD)	Immunological DDI (iDDI)	AI Approach
Mechanism	Enzyme inhibition/induction (e.g., CYP450); transporter competition	Three main domains: gut microbiome dysbiosis, systemic immunosuppression, and TME modulation	Multi-modal integration across clinical, genomic, microbiome, and spatial omics data
Temporal dynamics	Hours to days (drug half-life, metabolic clearance)	Weeks to months (immune priming, microbiome recovery, TME remodelling)	Time-series modelling (RNN/LSTM) for critical exposure windows; NLP for temporal extraction from clinical text
Context dependency	Predictable from drug properties; generally independent of clinical indication	Highly context-dependent: timing, indication, dose, and route	LLMs with RAG for context-dependent reasoning; knowledge graphs for mechanistic integration
Causal inference	Straightforward; reproducible in controlled PK studies	Limited by confounding by indication (“sick-user” bias); RCTs often ethically infeasible	ML adjusting for confounders; XAI (SHAP/LIME) for interpretability and counterfactual reasoning
Clinical decision support	Established rule-based alert systems in pharmacy software	No established CDSS; guidelines do not address iDDIs systematically	AI-based CDSSs with explainable outputs; SaMD frameworks evolving; no iDDI-specific system yet deployed

Abbreviations: *CDSS* clinical decision support system, *CYP450* cytochrome P450, *DDI* drug–drug interaction, *iDDI* immunological drug–drug interaction, *LIME* Local Interpretable Model-agnostic Explanations, *LLM* large language model, *LSTM* long short-term memory, *ML* machine learning, *NLP* natural language processing, *PD* pharmacodynamics, *PK* pharmacokinetic, *RAG* retrieval-augmented generation, *RCT* randomised controlled trial, *RNN* recurrent neural network, *SaMD* software as a medical device, *SHAP* SHapley Additive exPlanations, *TME* tumour microenvironment, *XAI* explainable artificial intelligence

## Discussion

This review integrates mechanistic, clinical, and computational perspectives to characterise iDDIs during ICI therapy. Collectively, the evidence highlights that iDDIs represent a distinct and clinically relevant class of interactions, mediated through host immune mechanisms rather than direct pharmacological effects.

However, the current evidence base remains largely retrospective and observational, limiting causal inference. As highlighted throughout this review, the interplay between confounding by indication and the temporal dynamics of drug exposure represents a major barrier to interpretation. For example, antibiotic-associated effects are highly dependent on timing, corticosteroid-associated effects are strongly influenced by indication (“indication-inversion”), and PPI–associated effects may reflect microbiome-mediated confounding. While Section "Immunological Drug–Drug Interactions with Immune Checkpoint Inhibitors" detailed these challenges, the integration of multi-modal AI pipelines—linking microbiome sequencing, longitudinal EHR data, and precise medication timing, offers a potential pathway to disentangle these overlapping effects. Despite consistent associations between several drug classes (including antibiotics, PPIs, and corticosteroids) and altered ICI outcomes, it remains unclear whether these relationships reflect true biological interactions or surrogate markers of patient complexity and disease severity. Accordingly, well-designed prospective studies and advanced analytical frameworks are required to move beyond association toward causal inference.

From an AI perspective, the field is emerging but rapidly evolving. As outlined in Section "Immunological Drug–Drug Interactions with Immune Checkpoint Inhibitors", AI approaches provide a complementary framework that maps naturally onto the challenges of iDDIs: NLP enables extraction of temporally sensitive medication exposures from unstructured clinical text; LLMs support context-dependent reasoning; ML facilitates outcome prediction in the presence of complex confounding; and explainable AI enables transparent clinical interpretation. Together, these methods form a pipeline from data extraction to clinical decision support. The addition of spatial and single-cell omics, metaproteomics, and knowledge graph-based integration (Section "Emerging Technologies: Omics and Knowledge Graphs") offers a further dimension, enabling the incorporation of molecular and mechanistic evidence into frameworks that have traditionally relied on clinical and administrative data alone.

However, several translational challenges must be addressed before these approaches can be routinely integrated into clinical practice. First, the lack of curated and annotated datasets specifically designed for iDDIs limits the development and evaluation of robust models [105]. Unlike traditional pharmacokinetic DDIs, immunological interactions are highly context-dependent and may be influenced by factors such as treatment timing, dosage, microbiome composition, tumour biology, and host immune status. These multidimensional interactions make predictive modelling inherently more complex. Second, a major research gap exists in the "triangulation" of multi-modal data: while existing studies often focus on microbiome composition or clinical outcomes in isolation, few integrate microbiome sequencing, clinical narratives, and high-resolution medication exposure data within a unified analytical framework. Third, many current AI applications in immunotherapy have been developed and validated within single-institution datasets, often without external validation, raising concerns regarding their generalisability across healthcare systems and patient populations [78]. Fourth, the “black box” nature of many AI models remains a major barrier to clinical adoption, particularly in high-stakes oncology decision-making [106]. Importantly, the context-dependent nature of iDDIs introduces unique risks for AI deployment. As demonstrated in Section "Immunological Drug–Drug Interactions with Immune Checkpoint Inhibitors", the clinical indication for a medication (e.g., corticosteroids for irAEs versus palliation) can invert its apparent prognostic effect. AI systems that fail to account for such context may generate misleading alerts and contribute to inappropriate withholding of necessary supportive therapies. Although XAI methods such as SHAP and LIME provide insight into model behaviour, their interpretation requires a level of statistical literacy that may not be universally available among clinicians.

A promising path forward lies in the integration of large-scale real-world data with AI-driven analytical frameworks. National population-level data resources, such as Australia’s Person-Level Integrated Data Asset (PLIDA), which links demographic, healthcare utilisation, and medication exposure data, demonstrate the potential value of large national datasets for studying immunotherapy outcomes in unselected populations [107–110]. When coupled with AI methodologies, these datasets enable modelling of longitudinal polypharmacy exposure, identification of critical windows of vulnerability during ICI treatment, and integration of real-world observational signals with mechanistic insights derived from preclinical and translational research. Future research should move beyond model development toward implementation science, focusing on whether AI-driven approaches can improve clinical decision-making, optimise supportive medication use, and enhance patient outcomes in real-world oncology practice**.**

## Conclusion

Immunological DDIs represent a clinically important and mechanistically distinct category of drug interactions that can significantly influence ICI outcomes. Their complexity extends beyond traditional PK or PD frameworks. These interactions are inherently dynamic, context-dependent, and mediated through host immune pathways, necessitating analytical approaches capable of integrating temporal, clinical, and biological complexity. As outlined in this review, iDDIs can be conceptualised across three principal mechanistic domains—gut microbiome disruption, systemic immunosuppression, and tumour microenvironment modulation—each with distinct clinical implications and analytical requirements. Artificial intelligence offers a promising pathway toward “predictive pharmacovigilance,” enabling detection, modelling, and interpretation of iDDIs across diverse data sources. However, current evidence remains largely proof-of-concept. Future research should prioritise prospective validation, multi-modal data integration, methodological standardisation, and demonstration of real-world clinical utility, alongside robust clinical governance frameworks, to ensure safe and effective integration of AI into immunotherapy practice.

## Key References

- Grice S, Olsson-Brown A, Naisbitt DJ, Hammond S. Immunological Drug–Drug Interactions Affect the Efficacy and Safety of Immune Checkpoint Inhibitor Therapies. Chem Res Toxicol. 2024;37(7):1086—1103. 10.1021/acs.chemrestox.4c00067.
  - ○ This is the foundational paper introducing the term "immunological DDIs" in the context of ICI therapy that underpins the conceptual framework of the present review.
- Crespin A, Le Bescop C, de Gunzburg J, et al. A systematic review and meta-analysis evaluating the impact of antibiotic use on the clinical outcomes of cancer patients treated with immune checkpoint inhibitors. Front Oncol. 2023;13:1075593. 10.3389/fonc.2023.1075593.
  - ○ The largest meta-analysis on antibiotics and ICI outcomes to date (107 studies). This study consolidates the evidence base for microbiome-mediated iDDIs and provides the key effect estimates cited throughout Section "Gut Microbiome–Mediated iDDIs: Antibiotics and Proton Pump Inhibitors".
- Lopes S, Pabst L, Dory A, et al. Do proton pump inhibitors alter the response to immune checkpoint inhibitors in cancer patients? A meta-analysis. Front Immunol. 2023;14:1070076. 10.3389/fimmu.2023.1070076.
  - ○ The most comprehensive PPI–ICI meta-analysis (41 studies, *n* > 20,000), reporting worse OS and PFS with concomitant PPI use across multiple tumour types. It provides the principal effect estimates for the PPI discussion in Section "Gut Microbiome–Mediated iDDIs: Antibiotics and Proton Pump Inhibitors" and highlights baseline/60-day pre-ICI exposure as the most clinically relevant window.
- Verheijden RJ, De Groot JS, Fabriek BO, Hew MN, May AM, Suijkerbuijk KPM. Corticosteroids for Immune-Related Adverse Events and Checkpoint Inhibitor Efficacy: Analysis of Six Clinical Trials. J Clin Oncol. 2024;42(31):3713—3724. 10.1200/JCO.24.00191.
  - ○ A post hoc analysis of individual patient data from six registrational trials demonstrating that peak corticosteroid dose—but not cumulative exposure—is independently associated with worse survival in ICI-treated patients. This is the strongest trial-level evidence supporting dose-dependent corticosteroid effects and informs the "indication-inversion" concept discussed in Section "Systemic Immunosuppression–Mediated iDDIs: Corticosteroids, Opioids, and Benzodiazepines".
- Yiu CH, Lau ECY, Le CTT, Lu CY. Leveraging Artificial Intelligence for Immune Checkpoint Inhibitor Safety: A Scoping Review of Current Applications. JCO Clin Cancer Inform. 2026;(10):e2500323. 10.1200/CCI-25-00323.
  - ○ The first scoping review systematically mapping current AI applications for ICI safety. It provides the evidence synthesis underpinning Section "Artificial Intelligence Approaches for Identifying and Managing Immunological DDIs" and highlights the gap between proof-of-concept tools and clinical deployment.

## Data Availability

No datasets were generated or analysed during the current study.
